# “*It seems impossible that it’s been made so quickly*”: a qualitative investigation of concerns about the speed of COVID-19 vaccine development and how these may be overcome

**DOI:** 10.1080/21645515.2021.2004808

**Published:** 2022-02-16

**Authors:** Poppy Brown, Felicity Waite, Michael Larkin, Sinéad Lambe, Helen McShane, Andrew J. Pollard, Daniel Freeman

**Affiliations:** a Oxford Institute for Clinical Psychology Training and Research, Oxford, UK; b Oxford Health NHS Foundation Trust, Oxford, UK; c Department of Psychiatry, University of Oxford, Oxford, UK; d Institute of Health and Neurodevelopment, Aston University, Birmingham, UK; e The Jenner Institute, Nuffield Department of Medicine, University of Oxford, Nuffield, Oxford, UK; f Department of Paediatrics, Oxford Vaccine Group, Oxford, UK

**Keywords:** Vaccine-hesitancy, Covid-19, qualitative, IPA, speed of development

## Abstract

The speed of COVID-19 vaccine development has been identified as a central concern contributing to hesitancy in acceptance. We conducted qualitative interviews to gain a greater understanding into these concerns and to identify what might address them. Twelve qualitative interviews were conducted with participants identifying as hesitant for COVID-19 vaccination and reporting concern about the speed of vaccine development. Interpretative Phenomenological Analysis (IPA) was used. Concerns about speed comprised the linked themes of i) difficulty understanding the pace, and, ii) worry about the implications for vaccine safety. Uncertainties concerning the pandemic led to a notable desire for credible and understandable information regarding the vaccines, which many participants felt was not available. Four routes to resolving uncertainty about whether to be vaccinated were identified. First, waiting for more information about the vaccines, such as about their contents and impact on transmission. Second, a growing perception that the vaccines must be safe given the large numbers already vaccinated. Third, viewing the vaccines as necessary – even if unappealing – for ending the pandemic. Finally, a feeling that there would be no choice but to have a vaccine. Examples of what might reduce hesitancy were given, including interviews with vaccine developers and knowing others of similar age having safely been vaccinated. The pace of development broke expectations set earlier in the pandemic. This was interpreted negatively due to a perceived lack of credible information. Most participants could envisage ways their concerns could be resolved, enough for them to have a vaccine.

## Introduction

In the Oxford Coronavirus Explanations, Attitudes, and Narratives Surveys (OCEANS) we have been developing the understanding of COVID-19 vaccine hesitancy in the UK in order to inform information provision. In OCEANS-II, concern about the speed of vaccine development was identified as one of four key beliefs about the vaccines that explained an extremely high proportion of the variance across the population in COVID-19 vaccine hesitancy.^1^ In OCEANS-III, messaging that directly addressed safety concerns about the speed of vaccine development reduced hesitancy in those reporting that they would delay taking a COVID-19 vaccine as long as possible or would never take one.^2^ In order to further refine messaging, our aim was to carry out in-depth interviews focussed on speed of development concerns with vaccine hesitant individuals.

To date there have been extremely few qualitative studies on COVID-19 vaccination attitudes. Typically, these have focussed on interviewing specific populations about hesitancy, such as adults with experience of homelessness^3^ or vaccine stakeholders in China.^4^ In one study interviews were conducted with an ethnically diverse sample of community leaders and influencers in Bradford in September to October 2020, finding that vaccine hesitancy could be attributed to three factors: safety (particularly due to how quickly the vaccines had been produced), hearing negative stories, and personal knowledge about health, diseases, and vaccines.^5^

Although suggested as a contributory factor to vaccine hesitancy in quantitative studies,^6^ to our knowledge there has been no in-depth investigation into people’s concern around the rapid speed of Covid-19 vaccine development, and, importantly, how this concern may be overcome. Our aim, therefore, was to conduct interviews with vaccine hesitant individuals reporting concern about the speed of vaccine development.

## Method

### Approach

The study used qualitative methods with a phenomenological focus, specifically Interpretative Phenomenological Analysis (IPA).^7^ Phenomenology is often framed as way of examining experience, but “experience” is often a useful, general shorthand^7^ for what might be more accurately described as “orientation.”^8^

That is, phenomenological analysis is concerned with understanding people’s orientation toward the salient features of their world and the individual meaning those features carry.

This finer distinction is important for the current study, where the focus is on understanding the meaning of something (vaccine development) which has become important to people, even if they do not have direct “experience” of it, and where it was purposively chosen to speak to respondents who have expressed concerns about it.

The lead author wrote a reflexive statement before conducting interviews and a bracketing interview was recorded. Consideration was given to the fact that the authors were approaching the topic from a primarily psychological perspective and that they had been engaged in thinking carefully about how the uptake of vaccines might be increased. The topic guide was piloted a number of times, and open, non-leading prompts to use during the interviews were formulated in order to minimize bias.

### Sampling

IPA is an approach which involves commitments to idiographic levels of analysis and context-sensitivity. In order to meet these commitments, informational depth is prioritized over breadth of representation.^9^ In IPA, sampling usually develops from a rationale about which dimension(s) of the sample should be homogenous. In the study, it was chosen to prioritize the participants’ perspectives on vaccination as a common feature, with an emphasis on understanding those with concerns, and especially those with worries about speed of development. We recruited our participants from respondents to the Oxford Coronavirus Explanations, Attitudes, and Narratives Survey II (OCEANS II),^1^ a participant group of over 5000 who had been quota sampled to be nationally representative for gender, age, ethnicity, income, and region. OCEANS-II was carried out in September and October 2020, before the UK vaccination programme had begun. We used scores on the Oxford COVID-19 Vaccine Hesitancy Scale^1^ to identify participants who reported uncertainty about whether to accept a COVID-19 vaccine, or who identified as strongly vaccine hesitant (i.e. would delay getting a vaccine for as long as possible), or who explicitly described themselves as anti-vaccination for COVID-19 and would refuse to have a vaccine. We invited participants to take part in an interview if they also reported thinking that the speed of the vaccine development meant the vaccines would be “bad” or “really bad” as well as “unsafe” or “really unsafe” (assessed on items six and seven of the Oxford COVID-19 Vaccine Complacency and Confidence Scale.^1^ Eight people per group (uncertain, strongly hesitant, anti-vaccination) were randomly selected to be contacted in the first instance and interviews were arranged with those who responded. Given this modest degree of homogeneity with regard to concerns about vaccination, and the more concentrated shared worry about speed of development, we then used further purposive sampling to ensure a mix of age and gender. Participants were included even if they had since accepted a COVID-19 vaccine, as this would give highly relevant information as to what factors had allowed participants’ concerns about speed to be overcome, enough to accept a vaccine. In total, 78 individuals were invited to participate in the study, with 17 volunteering to participate. We interviewed 12 participants. All participants who were invited to interview had given their permission in the initial survey to be re-contacted. Informed oral consent was recorded separately for each participant at the beginning of the interview. Ethical approval for the study was received from the Central University Research Ethics Committee (CUREC, reference: R71830/RE001).

### Participants

In this final participant group, participants ranged in age from 18 to 70 years old (mean = 46.33 years, SD = 17.58 years) and two-thirds were White (n = 8). Table 1 summarizes the participants’ demographic characteristics. Pseudonyms were assigned to all participants.

**Table 1.** Participant characteristics (n = 12)

Participant	Group	Gender	Age	Ethnicity	Total household income	Region in UK	Highest educational qualification	Current vaccination acceptance stance
1. Joseph	Strongly hesitant	M	66	White	£20-29,000	North West	Post-graduate qualification	Would accept now
2. Sean	Uncertain	M	43	White	£40-49,000	East	A Levels or equivalent	Uncertain
3. Ken	Anti-vaccination	M	34	White	£60-69,000	London	Higher education	Uncertain
4. Hannah	Strongly hesitant	F	39	White	<£15,000	East Midlands	As Levels or equivalent	Uncertain
5. Kate	Anti-vaccination	F	69	White	<£15,000	London	Higher education	Refused
6. Serena	Uncertain	F	18	White and Asian	£40-49,000	South East	A Levels or equivalent	Uncertain
7. Patricia	Uncertain	F	66	White	£40-49,000	East	A Levels or equivalent	Received
8. Reg	Anti-vaccination	M	70	Pakistani	£30-39,000	West Midlands	GCSEs or equivalent	Received
9. Danny	Uncertain	M	56	African	£60-69,000	London	Post-graduate qualification	Uncertain
10. Ayesha	Strongly hesitant	F	28	Pakistani	£40-49,000	North West	As Levels or equivalent	Received
11. Rupert	Strongly hesitant	M	40	White	<£15,000	West Midlands	GCSEs or equivalent	Uncertain
12. Lily	Uncertain	F	27	White	£40-49,000	Scotland	A Levels or equivalent	Would accept now

### Procedure

The study took place online in the UK. We developed a semi-structured guide with questions covering views on vaccines in general, views on the COVID-19 vaccine speed of development, participants’ initial reaction to either having been offered or hypothetically being offered a COVID-19 vaccine, and exploring drivers of increased and decreased confidence. The first author, who had previous experience training in and leading qualitative interviews, conducted interviews during March 2021 via Microsoft Teams (n = 3), or by telephone (n = 9) when a participant preferred. Interviews were audio recorded and transcribed verbatim. Consent was recorded verbally at the beginning of the interview. Interview length ranged from 37 minutes to 68 minutes (mean = 52.08 minutes, SD = 11.40 minutes).

### Analysis

We analyzed the transcripts using IPA. Four authors were involved in the analysis. The first author led detailed annotation of the transcripts, focusing on coding the claims and concerns of the participants, and then identifying patterns of meanings in those claims and concerns. She discussed the developing analysis with the wider authorship team, and then produced a case-level summary for each participant. PB, FW, ML, and DF reviewed the analytic work, and discussed the cases, to contribute to the thematic development for the cross-case analysis which is presented here. Considerable verbatim extracts are provided throughout given this is a key component of ensuring sensitivity to the raw data and thus minimizing any potential bias in analysis.^7^ Analysis was not separated by those self-describing as “anti-vaccination for Covid-19” (group 4) and those merely reporting uncertainty or hesitance (groups 2 and 3) given literature suggests that differences in beliefs regarding vaccination are primarily in strength rather than content.

## Results

At the time of the interview, six participants were uncertain of what their decision would now be when offered a COVID-19 vaccine. One participant (Kate) had been offered a vaccine and refused it. Three participants (Patricia, Reg, and Ayesha) had received a first dose of a Covid-19 vaccination, and a further two participants, Joseph, and Lily, were now intending to accept the vaccine when offered.

**Figure 1.**
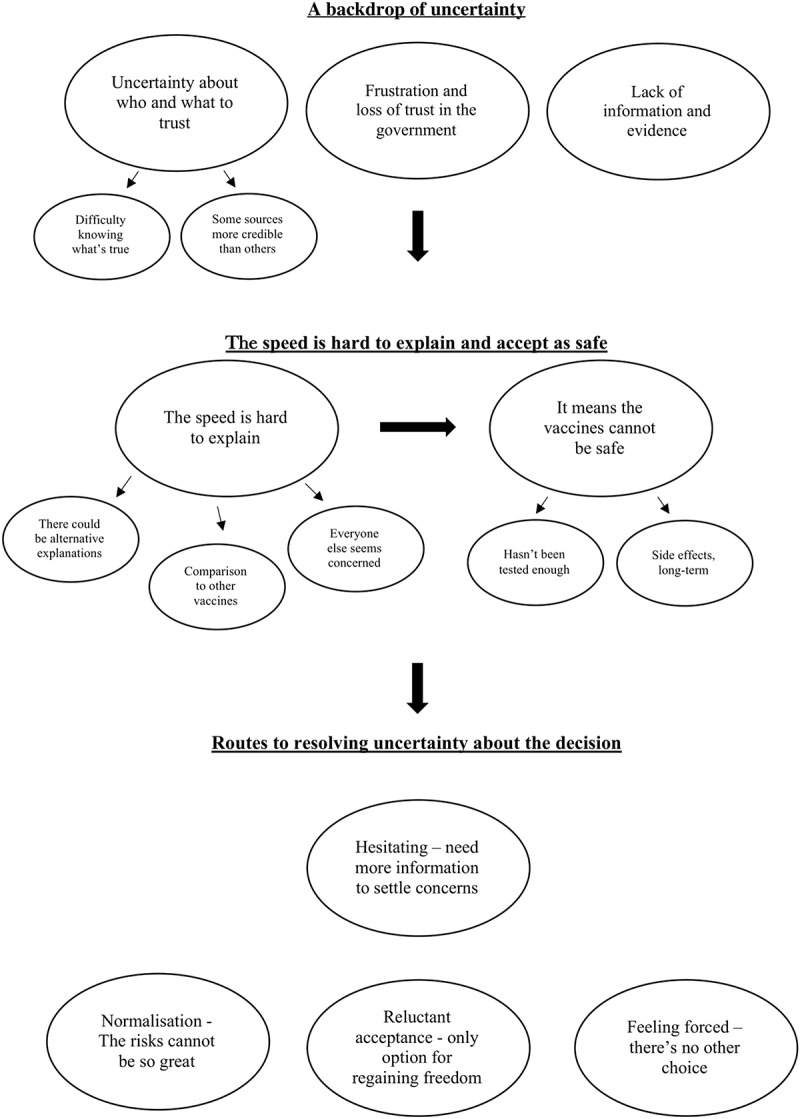
Diagram of results.

Three themes with 9 subordinate themes were identified (summarized in Figure 1). Thoughts concerning the vaccines occurred for the participants against a backdrop of uncertainty, a perceived lack of information, and a loss of trust in the government. A number of the participants wanted greater information about the vaccines but found it hard to know who and what could be trusted. Comparisons to vaccines for other conditions meant participants found the speed of development of the COVID-19 vaccines difficult to understand and explain. This difficulty led to uncertainties regarding the safety of the vaccine. Participants worried whether the vaccines could have been tested well enough in the time available, or whether there may be adverse side effects yet to be discovered. Six participants mentioned concerns over the potential impact on fertility and child development. Several routes to resolving uncertainty about whether to have a vaccine were considered by participants. Those who remained hesitant felt more information and evidence was required to increase their confidence. Nonetheless, nearly all participants found the number of people across the UK having a vaccine a source of reassurance that the risks could not be too great. Several participants also recognized the vaccines as the only way of coming out of the pandemic and regaining freedom, meaning they might accept it even if reluctant. Others felt they would have no choice but to have a vaccine due to the potential implementation of vaccine passports.

### A backdrop of uncertainty

The first theme relates to a contextual issue, which we include as important background for the substantive themes focussed directly on speed of development. Participants made sense of the speed of the vaccine-development, and the decision about whether to have the vaccine, against a backdrop of uncertainty (“*the entire pandemic has been very much like people not knowing what’s going on,”* Serena), lack of information (“*there are unanswered questions*,” Ayesha) and frustration with the government (“*we are being punished for the Government’s stupidity and arrogance*,” Rupert).

Participants’ uncertainty and desire for information concerned multiple aspects of the pandemic, including the severity of the virus (“*It’s sometimes hard to know how severe the pandemic is given the news keeps changing,”* Reg) and where the virus may have originated from (*“The virus could be manmade by the East, or the West, or by scientists wanting to make more money, we cannot know,”* Danny). While some such as Joseph and Sean had clear views about what makes information reliable and believable, such as *“peer-reviewed journals … the Lancet, the BMJ and the digest of journals”* (Joseph) or “*an independent body … Not somebody affiliated with the government or any scientific kind of company,”* (Sean), others found assessing credibility more challenging.

When I’m trying to look at this stuff or people are sharing PDFs and white papers and yellow papers and all kinds of documentation from apparently this scientist or that scientist, or this lab or that lab, I have no idea who these people are, if they are credible, who funds them, are they invested in a company that would benefit from this decision or that decision? … We just don’t know what is and what isn’t real. (Ken)

Ken’s difficulty with assessing the credibility of information appeared to be linked to a loss of trust in the government:

There’s a lack of trust in new establishment. I think if Boris Johnson told most people in the country today it was raining, they would look out the window. It doesn’t really matter what he’s saying, whether it’s right or not, people have just automatically got the assumption now that you’re just lying to me. (Ken)

Dissatisfaction with the government was echoed among a number of the participants, including those now in favor of the vaccines such as Joseph (*“The government, I believe, wish to use the vaccination programme as a means to promote their own position”*) and Ayesha (*“the Government now are actually being really unsupportive”*). On the other hand, three participants, Reg, Lily, and Danny, did speak positively of the government, acknowledging their efforts to manage a very difficult situation: *“I support the government … they are trying to do their best”* (Reg).

Two participants who had already made up their mind about the vaccines also reported being satisfied with the current level of information, in contrast to the rest of the participant group. For example, Lily said “*All the information I want is readily available,”* and Reg said “*Information is nice but I’m not too worried about having more*.” Joseph also noted that sometimes too much information could be a bad thing, fueling further uncertainty and confusion: “*I put myself into information-overload … ruminating over mountains of data wondering what to do.”*

Overall, it was clear that for the majority of participants, the uncertainties around the pandemic had led to a particular desire for credible and understandable information regarding the vaccine, which many of them felt was not available.

### The speed is hard to explain and accept as safe

Against this backdrop there were two key inter-linked concerns regarding the speed of vaccine development. Firstly, the speed was viewed as difficult to understand and explain. Secondly, this inexplicability led participants to view the speed as potentially having negative consequences for the safety of the vaccines.

In trying to explain the speed, nearly all participants made comparisons with other vaccines that had taken much longer to develop (“*Usually, vaccines seem to take longer*,” Lily; *“I’m no scientist but I always believed vaccines took years to make and get right and develop and test*,” Rupert). Such comparisons were made regardless of participants’ overall stance toward the vaccine. While some noted that extra resources and the severity of the situation had led to a swift development of this vaccine, for others, such as Hannah, this was not reassuring: “*Throwing money at something doesn’t make it better.”* Worries about the inexplicability of the speed were often exacerbated by social media and discussion with friends and family: “*there was a lot of talk about, “oh, this is quick,”* Patricia; “*There was a lot of people talking … why is it so quick?*” Reg. In order to explain the speed a number of participants considered whether corners had been cut during the vaccine development and testing stages: “*it just seems impossible that they could have completed these trials with the same standards as other trials,”* (Serena). This possibility was also considered by those planning to have the vaccine, such as Joseph (“*I think it was done too fast, and I think as a result the preparation wasn’t done”*). Several of those who were unsure about whether to have a vaccine also turned to alternative explanations and conspiracy thinking, including that the virus was manmade, and that the vaccines had already existed.

That was what people say, that it takes 15 years before they can discover vaccines. But this one has taken only 12 months, or less than 12 months … This was so much quicker now, so I have to think, “How is it going so much quicker?” I don’t know. I didn’t expect COVID-19 to happen before – is it manmade? When you plan something already, okay, let us plan COVID-19, plan for the medicine already. I don’t know, maybe that is why it is quicker. I don’t know (Danny)

This quote from Danny encapsulates the attempt to make sense of how the vaccines had been made so quickly and the lack of certainty about what kind of explanation is most plausible. The comparison between the two timeframes of 15 years and less than 12 months is particularly striking. For the most part other participants made a comparison between one year and several years (e.g. “*Usually it takes several years to develop a vaccine and they’ve done it in about a year*,” Sean). The larger contrast in timeframes considered by Danny may partly explain why he turned to alternative theories to explain the speed of development.

Concerns about the speed of development impacting the safety of the vaccines primarily revolved around a lack of wide-spread testing (“*you can’t possibly test this on every single person in society to know whether or not it’s going to work for everybody,”* Ken) and the potential long term side effects that could not have been identified in the timeframe that had passed, even if testing had been very wide-spread.

There was mass testing but it was over a shorter period of time than other vaccines had been. So, I think there could be so many side-effects in the long term that we don’t know about. And we don’t have time to find out about them … if I was to have children, I would be scared if it had an effect on them. (Serena)

Serena’s quote suggests that even if the speed was explicable in the circumstances, and the testing wide-spread enough, there is still an issue with regards to knowing about side effects in the long-term. This issue was echoed across many of the interviews: “*what will this vaccine do to you long-term?”* (Rupert); “*no-one knows the long-term effects. It’s not been tested on anyone for that”* (Hannah). Five further participants, both male and female, reported either previously or currently sharing Serena’s more specific concern about whether the vaccines could impact fertility or child development. These participants varied as to whether their concern was for their own fertility or that of friends and family. Understandably, however, most of these participants were the ones who felt most uncertain about having the vaccine, and also tended to be younger in age. Interestingly, impact on fertility was the only specific adverse effect that participants reported being worried about. With this exception, concern about side effects was very general and not limited to particular types of effect on health.

There were two notable exceptions to concerns about the quick development of the vaccine. Firstly, for Ayesha, the vaccines had in fact been developed much more slowly than she had originally expected given all the funding and numbers of scientists working on it: “*I expected something like three or four months … I didn’t really expect it to be so long*.” The apparent delay in production had initially made her concerned that safe development of a vaccine was not going to be possible, a concern that subsided upon approval of the vaccines.

Secondly, for Kate the speed of vaccine development wasn’t a relevant question. Kate’s view was that the Covid-19 vaccines are not real vaccines but are instead “*a human gene experiment designed to kill people.”* She strongly endorsed a conspiracy belief that the vaccines had been in development for some time by Bill Gates and other members of the elite.

With these two exceptions, there was a lot of consistency in the concerns participants raised about the speed of the vaccine development. Unsurprisingly, fear of corners having been cut and negative side effects were endorsed to a greater extent among those who were still undecided about having the vaccine.

### Routes to resolving uncertainty

In spite of the concerns about speed, Kate was the only participant who reported having been offered a vaccine and refusing it. Four routes for resolving uncertainty in order to help reach a decision were considered by participants.

Firstly, those who were still unsure about whether they would accept a vaccine described how their confidence and certainty about having a vaccine would be increased if they had access to better and more information: “*information needs to be more readily available and accessible”* (Serena). Apart from a desire to know about any side effects, several participants also wanted to know exactly what is contained in the vaccines: “*We don’t even know what’s contained in this vaccine. Where they get it from.”* (Danny). Several also wanted to know whether the vaccines impact transmission, including those such as Joseph and Lily who were nonetheless already planning to have the vaccine. Some participants were very specific in suggesting the format of the information they wanted. For instance, Hannah suggested using door-to-door leafleting, and Ken described the importance of media outlets like podcasts: “*Most people are getting their information from longform media like podcasts. I think it would be really beneficial if there was a bit more of an active outreach into different forms of media.”*

A second route to resolving uncertainty was through acknowledgment that given the many millions of people in the UK who have already had the vaccine, the potential unknown risk of the vaccines must have decreased over time. This sense of normalization of the vaccine, and a feeling that it must be safe if so many people were having it, was a significant driver of increased confidence in the vaccines both in those who had already received it, such as Patricia, (“*I felt more positive about it because there were so many people that were having it done”*) as well as those who were still uncertain such as Sean (“*I’m a bit more accepting of it now, now more people have had the vaccine”*).

A third route to resolving uncertainty was a recognition that regardless of feelings about speed and safety, the vaccines may be the only way out of the pandemic, and thus also the only way of regaining freedom: “*It seems really necessary to help stop this pandemic”* (Serena). For a number of participants this was seen as the primary benefit of the vaccine, and an important factor that makes being vaccinated worth the risk of side effects. For example, Hannah, who was otherwise undecided about taking a vaccine said *“If someone said to me today, you can have the vaccine today and you can … go where you want to go tomorrow, I would have it done now*.”

Finally, there was also significant discussion over the potential for “vaccine passports,” with several participants feeling that eventually there will be no choice but to have the vaccine: “*basically you’re not going to really be left with any choice but to have it”* (Sean). Many believed that once restrictions eased you wouldn’t be able to travel abroad, or potentially even enter restaurants and other places without proof of having had a vaccine. There was conflict both between and within participants as to whether such restrictions would be fair or not: “*I do think it’s quite a good way of convincing people in general … but it does seem quite manipulative”* (Serena).

Nearly all participants considered multiple of these routes during their interview.

If I had to have it so that I could have a social life then I would have it … . It’s just … it seems a bit forced. Does that make any sense? Over the last year as well, you’ve got people that question vaccination and how quick it’s come about and you’re sort of declared an anti-vaxxer and I don’t think that’s the case at all. I think people are just generally worried about the fact that they do not know what they’re putting in their body.

Then, again, on the flip side of that how many people … we are now 20 million vaccinated? And I’ve not heard of anyone dropping dead. So, it must be working. (Rupert)

Rupert’s quote helps to illustrate the complexity of the decision-making process. While he states that he would have a vaccine if it meant he could regain his social life, he also dislikes the extent to which it feels the vaccines are being forced on him. As with several other participants he mentions the issue of people wanting to know the contents of the vaccines and how that will affect their bodies and feels that this desire for further information should not mean that you are labeled as an “anti-vaxxer.” Finally, he considers how given the numbers of people who have now been vaccinated without any significant negative effects, then the vaccines must surely be safe and effective. In spite of this, he remains uncertain about whether he would have a vaccine (“*I’m not for it; I’m not against it”*) whereas for others this had been a deciding factor (“*after a while when a lot of people got the vaccination and then there wasn’t much bad news coming through, so I changed my mind*,” Reg).

This third theme was arguably where there was most variance between but also within participants, highlighting the conflicting thoughts and emotions that a number of the participants were experiencing. Undoubtedly, those who had decided to have a vaccine fell most strongly within the normalization pathway, whereas those who remained most uncertain fell most strongly within the hesitating and feeling forced themes.

## Discussion

This study reports the first in-depth qualitative analysis of one of the key contributors to COVID-19 vaccine hesitancy: concern about the speed of vaccine development. Participants thought about the speed of vaccine development within a context of feeling uncertain about many aspects of the pandemic. Combined with diminishing levels of trust in the government, this led participants to question the credibility and validity of information regarding the vaccine, and in some cases to consider alternative explanations for its fast development. The significance of wider trust, mistrust, and uncertainty in influencing thoughts about the vaccines is in line with findings from other studies. In their analysis of 20 interviews with residents in Bradford, Lockyer and colleagues^5^ described how the “more confused, distressed and mistrusting participants felt about their social worlds during the pandemic, the less positive they were about a vaccine.” Similarly, a large cross-sectional survey found that higher trust in information given by government sources was associated with a higher likelihood of COVID-19 vaccine acceptance.^10^

Taking this context into account helps to explain the extent of concern about the speed of vaccine development. In the UK it had been repeatedly stated at the start of the pandemic that vaccines typically take many years to develop, thus one for COVID-19 should not be expected soon and people should adhere to immediately available measures, such as social distancing. This messaging was clearly difficult for participants to reconcile with multiple vaccines then being developed, tested, and approved, in less than 12 months.

It was also clear, however, that between vaccine approval in December 2020 and taking part in the interviews in March 2021, several participants’ concerns about the safety of the vaccines had largely subsided. This is reflected in the results of OCEANS-III, in which it was found that the proportion of the population who were vaccine hesitant had fallen from 27% to 17%.^2^ The biggest driver of reassurance was knowing how many other people in the UK had received a vaccine. While this could provide little reassurance about long-term side effects, it increased confidence that in the immediate term the vaccines are likely to be a safe and an important way to end restrictions due to the pandemic. It is also of note, however, that these interviews were conducted before the emerging reports of blood clots as a potential side effect of the AstraZeneca Covid-19 vaccine. Therefore, concerns raised about potential side effects during the interviews were not based on any adverse effects widely discussed in the media.

It was clear that participants felt current information access was not good enough. To reach those who engage less with typical news sites, information in the form of podcasts, leaflets, TV debates, and social media advertisements may be important. This is in line with Chadwick and colleagues,^11^ who suggest that direct contact through post, workplace, or community structures is important for reaching those who avoid the news, given their finding from OCEANS-II that the tendency to avoid typical news sources and take a “news finds me” attitude was associated with online discouragement of taking the COVID-19 vaccine. Hearing from researchers, popular scientists, and the vaccine developers themselves was also described as a strategy that would increase the confidence of the participants. Attention should also be paid to the types of questions typically asked by those vaccine hesitant, such as the contents of vaccines and how they are similar or different to existing vaccines. Consistent messaging, when possible, and careful means to address misconceptions and myths is also important for reducing the likelihood that people turn to alternative explanations and conspiracy theories to explain discrepancies in information. Changing information comes with increased cognitive load, making it more important for information to be easily accessible, clear, and supported by a range of sources.

Independence as a sign of credibility, and the dislike of mixing politics with science and health advice, were also discussed by multiple participants. This implies that a greater reliance on scientific institutions such as universities and health authorities to distribute information might be helpful. This is in line with a recent study that found that fact-checking labels for vaccine misinformation that were attributed to universities and health institutions were viewed more positively and as more trustworthy than other sources.^12^

Knowing how many other people had taken a vaccine was one of the most significant drivers of increased confidence in the vaccine. For some participants, however, it was only reassuring if they knew others of a similar demographic background who had had the vaccine. Normalizing the vaccines within local communities may therefore be helpful, to increase the likelihood that this variable can work as a driver of vaccine uptake.

There are limitations to the study. Firstly, the sample is largely self-selecting. Those who took part were willing to first complete a survey about the pandemic and associated vaccines, and then to take part in an interview. We may therefore have captured a group of people with particularly strong views concerning the vaccine and cannot know how representative the views presented are of the wider population. Secondly, while the sample could be considered reasonably large for an IPA study, it is obviously small in terms of understanding how the UK population thinks about the vaccine. Thirdly, we did not provide to participants as probes any formal explanations for the speed of development in order to test what kinds of information might change participants’ minds.

Finally, while the study does give us insight into the factors shaping people’s thoughts and concerns about the vaccine, we cannot have complete confidence about how this will impact actual behavior for those participants who had not yet been offered a vaccine. For these participants the questions were answered by considering a hypothetical scenario where they were offered a vaccine now, rather than by direct experience, which would be more typical for IPA. A follow up study would be required to measure actual behavior. Nonetheless, the study gives rich insight into how the participants’ oriented themselves with regards to the vaccines, and what factors are shaping their worries and concerns about the speed of development.
